# The Ever-Increasing Array of Novel Inborn Errors of Immunity: an Interim Update by the IUIS Committee

**DOI:** 10.1007/s10875-021-00980-1

**Published:** 2021-02-18

**Authors:** Stuart G. Tangye, Waleed Al-Herz, Aziz Bousfiha, Charlotte Cunningham-Rundles, Jose Luis Franco, Steven M Holland, Christoph Klein, Tomohiro Morio, Eric Oksenhendler, Capucine Picard, Anne Puel, Jennifer Puck, Mikko R. J. Seppänen, Raz Somech, Helen C Su, Kathleen E. Sullivan, Troy R. Torgerson, Isabelle Meyts

**Affiliations:** 1 Garvan Institute of Medical Research, Darlinghurst, Sydney, New South Wales 2010 Australia; 2 Faculty of Medicine, St Vincent’s Clinical School, UNSW Sydney, Sydney, NSW Australia; 3 Department of Pediatrics, Faculty of Medicine, Kuwait University, Kuwait City, Kuwait; 4 Laboratoire d’Immunologie Clinique, d’Inflammation et d’Allergy LICIA Clinical Immunology Unit, Casablanca Children’s Hospital, Ibn Rochd Medical School, King Hassan II University, Casablanca, Morocco; 5 Departments of Medicine and Pediatrics, Mount Sinai School of Medicine, New York, NY USA; 6 Grupo de Inmunodeficiencias Primarias, Facultad de Medicina, Universidad de Antioquia UdeA, Medellin, Colombia; 7 Laboratory of Clinical Immunology & Microbiology, National Institute of Allergy and Infectious Diseases, National Institutes of Health, Bethesda, MD USA; 8 Dr von Hauner Childrens Hospital, Ludwig-Maximilians-University Munich, Munich, Germany; 9 Department of Pediatrics and Developmental Biology, Tokyo Medical and Dental University, Tokyo, Japan; 10 Department of Clinical Immunology, Hôpital Saint-Louis, APHP, University Paris Diderot, Sorbonne Paris Cité, Paris, France; 11 Study Center for Primary Immunodeficiencies, Necker Hospital for Sick Children, APHP, Paris, France; 12 Laboratory of Lymphocyte Activation and Susceptibility to EBV, INSERM UMR1163, Imagine Institute, Necker Hospital for Sick Children, Paris University, Paris, France; 13 Laboratory of Human Genetics of Infectious Diseases, INSERM U1163, Necker Hospital, 75015 Paris, France; 14 Imagine Institute, University of Paris, 75015 Paris, France; 15 Department of Pediatrics, University of California San Francisco and UCSF Benioff Children’s Hospital, San Francisco, CA USA; 16 Adult Immunodeficiency Unit, Infectious Diseases, Inflammation Center and Rare Diseases Center, Childrens Hospital, University of Helsinki and Helsinki University Hospital, Helsinki, Finland; 17 Pediatric Department and Immunology Unit, Sheba Medical Center, Tel Aviv, Israel; 18 Division of Allergy Immunology, Department of Pediatrics, Childrens Hospital of Philadelphia, University of Pennsylvania Perelman School of Medicine, Philadelphia, PA USA; 19 Allen Institute for Immunology, Seattle, WA USA; 20 Department of Immunology and Microbiology, Laboratory for Inborn Errors of Immunity, Department of Pediatrics, University Hospitals Leuven and KU Leuven, 3000 Leuven, Belgium

**Keywords:** Inborn errors of immunity, immune dysregulation, primary immunodeficiencies, autoinflammatory disorders

## Abstract

The most recent updated classification of inborn errors of immunity/primary immunodeficiencies, compiled by the International Union of Immunological Societies Expert Committee, was published in January 2020. Within days of completing this report, it was already out of date, evidenced by the frequent publication of genetic variants proposed to cause novel inborn errors of immunity. As the next formal report from the IUIS Expert Committee will not be published until 2022, we felt it important to provide the community with a brief update of recent contributions to the field of inborn errors of immunity. Herein, we highlight studies that have identified 26 additional monogenic gene defects that reach the threshold to represent novel causes of immune defects.

## Introduction

Inborn errors of immunity (IEI) are generally considered to result from monogenic germline defects that manifest as increased susceptibility to severe and/or recurrent infectious diseases, autoimmune or autoinflammatory conditions, atopic manifestations, and hematopoietic or solid tissue malignancies [1]. Over the past decade, the discovery of new IEIs has been occurring at an impressive rate. Indeed, the 2011 biennial update published by the IUIS Committee update listed 191 IEIs; this number increased to 430 in the 2019 update [2, 3]. This near-exponential increase in gene discovery is being driven by the accessibility and affordability of next-generation sequencing, and the efficient application of these technologies to elucidate the molecular etiology of unsolved cases of IEIs that are likely to result from single-gene defects [4].

Over the last 12 months, we have witnessed the ongoing rapid identification, and occasionally detailed molecular, biochemical, and cellular characterization, of genetic variants that cause, or are at least associated with, human diseases impacting host defense or immune regulation. Here, we will summarize reports on variants detected in 26 genes which we consider represent novel IEI (Table 1). Many additional genetic variants have been reported recently. However, those listed here have been adjudicated by the IUIS Committee to meet the strict criteria to be considered disease-causing [57]. These criteria include:

1. The patient’s candidate genotype is monogenic and must not occur in individuals without the clinical phenotype;
2. Experimental studies must indicate the genetic variant impairs, destroys, or alters expression or function of the gene product;
3. The causal relationship between the candidate genotype and the clinical phenotype must be confirmed via a relevant cellular phenotype, including—where possible—rescue of a functional defect by reconstitution with the wild-type gene, or via a relevant animal phenotype [57].

**Table 1:** Newly validated inborn errors of immunity

**Novel gene defects underlying IEI—2020**								
**Genetic defect**	**Inheritance/mechanisms**	**T/NK cells**	**B cells**	**Ig levels**	**Clinical features, cellular defects, and evidence of variant pathogenicity**	**Table (for classication)**^**[2, 3]**^	**Refs**	
***PAX1*** (8 patients, 3 families; 2 papers with overlapping patients)	AR (LOF)	• T^−^B^+^NK^+^ SCID • Severe T lymphopenia, low TRECs,	• Normal	• ~ Normal IgM, low IgA, normal to ↑ IgE,	• Omenn’s-like syndrome (erythroderma, lymphocytosis, eosinophilia, ↓ proliferation to PHA, severe/recurrent infections), • No thymus, T cell deficiency not corrected by HSCT despite donor chimerism • Also: otofaciocervical syndrome type 2 (OTFCS2) **Validation** • Reporter assays established hypomorphic LOF of mutant alleles • Patients’ derived iPSCs differentiated to thymic epithelial cells demonstrated abnormalities in thymic epithelial progenitors, consistent with failure of HSCT to reconstitute T cells • Similar to mouse model	Table 1 Subtable 1	[5, 6]	
***SLP76*** (1 patient)	AR (LOF)	• T cells reduced, • ↓↓ CD4+, ↑ CD8+ T cell proportions • Low naïve, ↑ T_CM_ CD4+ T cells; • CD8+ T cell mostly clonally expanded T_EMRA_ • Low TRECs • NK normal numbers	• Normal numbers but ↓ class-switched memory and transitional B cells, • ↑ naïve and immature B cells	• High IgM, low IgA	• Combined immunodeficiency, • Early-onset skin abscesses, rash recurrent infections, autoimmunity, • Neutrophil dysfunction, platelets dysfunction • ↓ T cell proliferation to PHA, anti-CD3/CD28 stimulation, partially restored by IL-2 • ↓ NK cell degranulation • ↓ Actin polymerization **Validation** • Generation of SLP76-deficient Jurkat T cells; expressing WT or mutant *SLP76* allele, mimicking patient cellular phenotypes; • Partial rescue of some of the functional defects by expression of WT *SLP76*	Table 1 Subtable 1	[7]	
***MCM10*** (1 patient)	AR (LOF)	• Mild lymphopenia • ↓ T_CM_, T_EM_ cells • ↓↓↓ NK cells (↑ CD56^bright^, nearly absent CD56^dim^ mature NK)	• ↓ B cells	• slightly ↓ IgG, normal IgM/A	• Severe (fatal) CMV infection • HLH-like (based on biomarkers, not clinical features • Phenocopies *GINS1* and *MCM4* deficiencies • ↓ NK function **Validation** • Detailed functional analysis of *MCM10* variant in primary fibroblasts, transfectants, iPSC, and CRISPR/Cas9-gene-edited NK92 human NK cell line • assessed mutant allele on NK cell development in humanized mice reconstituted with CD34^+^ HSC generated from the patients or healthy donor IPSCs	Table 2 Subtable 2	[8]	
***IL6ST*** (gp130; 12 patients, 8 families)	AD (DN)	• Normal T cell numbers • ↑ naïve CD4 and CD8 T cell proportions • ↓ T_CM_ CD4+ and CD8+ T cells and T_EM_ CD8 T cells; • ↓ MAIT, Tfh cells, • ↑ Th2, • Low to normal NK cell counts	Normal numbers of B cells, low memory B cell proportions	• Normal/low IgG, A, • Normal IgM, • Hyper IgE. • vaccine IgG normal	• HIES – STAT3-like; • Dermatitis/eczema, eosinophilia, recurrent skin infections, pneumonia, bronchiectasis, pneumatoceles with severe secondary pulmonary aspergillosis, connective tissue defects (scoliosis, face, joints, fractures, palate, tooth retention) • Phenocopies aspects of IL6R and IL11R deficiencies (due to unresponsiveness to these cytokines) **Validation** **•** LOF and DN alleles shown by overexpression in GP130 deficient HEK293T cells; • Impaired GP130/STAT3 signaling (mostly downstream of IL-6) in patients’ fibroblasts and leukocytes	Table 2 Subtable 5	[9]	
***IL6ST*** (complete deficiency; 6 patients, 4 families)	AR (LOF)	ND (death in utero or in neonatal period occurred for most affected individuals)			• Fatal Stuve-Wiedemann-like syndrome; skeletal dysplasia, lung dysfunction, renal abnormalities, thrombocytopenia, dermatitis, eczema • Defective acute phase response • Complete unresponsiveness to IL-6 family cytokines **Validation** **•** Complete LOE (for one allele) and LOF for two alleles tested by overexpression in GP130 deficient HEK293T to all cytokines tested of the IL-6 family. • Effects of variants well-characterized, including a partial rescue of patient amniocytes	Table 2 Subtable 5	[10, 11]	
***FNIP1*** (6 patients; 5 families)	AR (LOF)	Mild T cell lymphocytosis	B cell lymphopenia (absent/low; BM block [few immature B cells])	agamma/hypogammaglobulinemia	• Early onset recurrent infections (sinopulmonary) • Bronchiectasis • Congenital heart defects (e.g., hypertrophic cardiomyopathy) • Variable neutropenia (severe or intermittent) Crohn disease (one patient) • Developmental delay • Increased AMPK activity **Validation** • Almost recapitulates the mouse model [12, 13]	Table 3 Subtable 1	[14, 15]	
***PIK3CG*** (2 patients; 2 families)	AR (LOF)	• Normal CD4, • ↓ Treg, ↓ CD8	• Normal but ↓ memory B cells	• Hypogamma • Intact vaccine responses	• Cytopenia/lymphopenia, eosinophilia, lymphadenopathy, splenomegaly, • recurrent infections • HLH-like; ↑ inflammatory markers **Validation** • ↓ T cell proliferation, activation in vitro • Cellular defects recapitulated in *PIK3CG* targeted Jurkat T cell line and *Pik3cg* ko mice	Table 3 Subtable 2	[16, 17]	
***CTNNBL1*** (1 patient)	AR (LOF)	↓ T cells	• Reduced memory B cells; • Impaired CSR, SHM	• Progressive severe hypogamma	CVID, autoimmune cytopenias, hypogamma, recurrent infections, hyperplastic GC’s; (mutation reduces binding of CTNNBL1 to AID, resulting in less nuclear translocation of AID) **Validation** • detailed functional analysis of *CTNNBL1* variant in EBV B lines, and engineered RAMOS cell lines; • mutant allele reduces the binding of CTNNBL1 to AID, with impaired nuclear translocation of AID; • defective SHM rescued in mutant Ramos cells by WT *CTNNBL1*	Table 3 Subtable 3	[18]	
***TNFSF13*** *(*APRIL; 1 patient)	AR (LOF)	• Normal T/NK cells	Normal total B cell counts, ↑ IgM+ marginal zone /↓ sw memory B cells, ↓ blood plasmablasts	• Hypogamma	CVID, chronic but mild infections **Validation** • LOE and LOF allele, • Functional analysis of the variant in PBMCs and overexpression; • Impaired function of iPSC-moDC in promoting B cell differentiation could be rescued with exogenous APRIL	Table 3 Subtable 3	[19]	
***SOCS1*** (15 patients; 10 families)	AD (by haploinsufficiency)	• ↓CD4, CD8 T cells	• Predom naïve B cells • ↓ sw memory, ↑ CD21lo B	• ↓ IgM, G, A but protective specific Abs; • ANA’s, autoAbs • ↑ serum BAFF	• Recurrent bacterial infections, • Severe multisystemic autoimmunity (flared in context of infection-induced inflammation), ITP, AIHA, SLE, GN, hepatosplenomegaly, psoriasis, arthritis, thyroiditis, hepatitis • Evan’s syndrome • 1 patient developed COVID19/MIS-C • neutropenia, lymphopenia • incomplete penetrance **Validation** • ↑ pSTAT1, ↑ type I/II IFN signature • Reporter assays confirmed impaired inhibition of mutant SOCS • Jakinib effective in vitro and in vivo.	Table 4 Subtable 4	[20–22]	
***TET2*** (3 patients, 2 families)	AR (LOF)	• ↑ DN T cells • ↓ Th1, Th17, Tfh cells	• ↓ Memory B cells • Impaired B cell diff in vitro to plasma cells	• Variable (hyper−/ hypogamma, ↓ response to pneumococcal vaccine	• ALPS-like (↑ sCD25, sFasL, IL10) • Recurrent viral infections, lymphadenopathy, hepatosplenomegaly, autoimmunity (cytopenias, B cell lymphoma (EBV+ HL-like) • Failure to thrive, developmental delay • EBV viremia • DNA hypermethylation • Defective FAS-mediated apoptosis	Table 4 Subtable 7	[23]	
***CEBPE*** (3 patients, 1 family)	AR (GOF)	• Mild lymphopenia •↓ naive, ↑ T_EMRA_ cells	ND	ND	• Recurrent abdominal pain, aseptic fever, systemic inflammation; abscesses, ulceration, infections; mild bleeding diathesis • Autoinflammasome activation/↑ IFN gene expression (autoinflammation, immunodeficiency, neutrophil dysfunction) **Validation** • homozygous *CEBPE* variant affecting DNA binding domain, associated with autoinflammation/immunodeficiency • showed altered chromatin occupancy of mutant CEBPE, and transcriptional changes, in patient cells targeting inflammasome and IFN-related genes	Table 5 Subtable 2	[24]	
***TBX21*** (T-bet; 1 patient)	AR (LOF)	• Normal %’s total, CD4 and CD8 T cells, naïve and memory subsets, low frequencies of NK cells • ↓ CXCR3^+^ CCR6^−^ Th1 cells, CXCR3^+^ Tfh and CXCR3^+^ Treg CD4^+^T cells • ↑ immature NK • ↓ iNKT, MAIT, Vδ2^+^ γδ T cells	• Normal	• Normal	• MSMD • ↓IFN-γ and TNF-α production by T cell subsets (γδ T cells, MAIT cells, iNKT cells, NK cells,, Vδ2^+^γδ, Vδ1^+^γδ, and CD4^+^ T cells **Validation** • Biochemical and molecular analysis established the impact of this variant on Tbet function; • Impaired production of IFNγ, TNFα by *TBX21*-mutant naïve CD4+ T cells under Th1 polarizing cultured in vitro • Tbet-dependent functions restored in patient cells and cell lines by WT *TBX21*	Table 6 Subtable 1	[25]	
***IFNG*** (2 patients [cousins])	AR (LOF)	• Normal frequencies of T and NK cells; • ↑ proportions of naive CD4+ and CD8+ T cells; • ↓ frequency of invariant iNKT	• Normal B cell frequencies; • ↓ memory B cells (↓ IgA^+^ / IgM^+^, ↑ IgG^+^ memory B cells)	• Normal	MSMD/BCG-osis • No IFN-γ producing cells **Validation** • LOE and LOF allele • Biochemical and molecular analysis established impact of this variant on IFN-γ production from patients’ cells • No IFN-γ production by T and NK cells; • Impaired IFN-γ production by patient-derived Herpesvirus *saimiri*–immortalized T cells restored by introduction of WT *IFNG*	Table 6 Subtable 1	[26]	
***NOS2*** (1 patient)	AR	• ↓ CD4+ T cells; • ↓ NK cells (mostly all immature cells) • normal CD8+ T	↓ B cells	ND (specific Ab levels normal)	• Severe susceptibility to CMV-induced disease; fatal • Pneumocystis pneumonia secondary to CMV • Apparent intact responses to infection with other herpes viruses (EBV, VZV, HSV) **Validation:** • Confirmed functional defect in transfected cells; truncated NOS2 failed to induce nitrous oxide **•** Recapitulates susceptibility of *Nos2* deficient mice to murine CMV infection [27] (these mice are also susceptible to numerous other pathogens)	Table 6 Subtable 3	[28]	
***SNORA31*** (5 patients, unrelated)	AD	• Normal	• Normal	• Seropositive for IgG against many viruses	• Forebrain herpes simplex virus-1 (HSV-1) encephalitis **Validation:** susceptibility of human pluripotent stem cell (hPSC)–derived cortical neurons from patients or hPSC-derived neurons from healthy donors but engineered to express variant *SNORA31* to HSV1 infection, corrected by exogenous IFN-β • Incomplete penetrance	Table 6 Subtable 4	[29]	
***ATG4A*** (1 patient)	AD	Normal	Normal	Normal	• Mollaret’s meningitis (recurrent lymphocytic meningitis) due to HSV2 • History of multiple episodes of meningitis; HSV2^+^ **Validation** • Impaired HSV2-induced autophagy → increased viral replication and apoptosis of patient fibroblasts • These defects were rescued by introduction of WT *ATG4* or *LC3B2* into patient fibroblasts	Table 6 Subtable 4	[30]	
***MAP1LC3B2*** (1 patient)								
***MAPK8*** (3 patients, 1 family)	AD (haploinsufficiency)	• Normal total T cells, CD4^+^ and CD8^+^ T subsets, NK cells • ↓ Th17 cells	• Normal total B cells and subsets	• Normal	• Chronic mucocutaneous candidiasis (CMC) • Connective tissue disorder (similar to Ehlers-Danlos syndrome) • ↓ Th17 cells ex vivo, in vitro • ↓ Responses of fibroblasts to IL-17A, IL-17F • ↓ c-Jun/ATF-2-dependant TGF β signaling **Validation** • *MAKP8* variant LOE in HEK293 T, heterozygous patients’ cells (↓ Th17 cells ex vivo, in vitro; ↓ fibroblast responses to IL-17A, IL-17F; ↓ TGFβ signaling) • Defective responses of fibroblasts restored by WT MAPK8	Table 6 Subtable 6	[31]	
***LSM11*** (2 siblings, 1 family)	AR (LOF)	Not reported			• Aicardi-Goutieres syndrome (type 1 IFN-opathy)	Table 7 Subtable 1	[32]	
***RNU7****–1* (16 patients, 11 kindreds)								
***CDC42*** (15 patients; numerous kindreds reported in 7 papers)	AD	• Normal/decreased T cell numbers, • normal %CD4/CD8 but skewed differentiation	• Normal/B-lymphopenia	• Variable (↑↓) IgM, G, A, E	• Neonatal onset: pancytopenia, fever, rash, hepatosplenomegaly, systemic inflammation, myelofibrosis/proliferation, HLH, multisystemic inflammatory disease. • ↑ serum levels of IL1, IL18, IFN-γ, ferritin, sCD25, CRP etc. • Recurrent GIT/RT infection; • Neurodevelopmental delay, FTT • Mutation affects actin function; • Treated with Anakinra/ IFN-γ mAb ↓NK function (cytox),	Table 7 Subtable 1	[33–39]	
***STAT2*** *(GOF,* 3 patients; all deceased; 2 additional deceased sibs but not genotyped; 2 unrelated families)	AR (GOF)	• Low frequency of NK, ↑ frequency of T cells (esp naïve), normal NK degranulation	• Total B cell frequencies within age-matched controls’ ranges • slight ↑ transitional and naïve B cells %‘s	• Low/normal	• Severe fatal early-onset autoinflammation (skin ulceration, fever, seizures, intracranial calcification, multiorgan dysfunction, abnormal neurodevelopment; phenocopy of USP18 deficiency) • ↑ serum IFN-α, IL6, TNFα • IFN-opathy gene signature (impaired regulation of late cellular responses to type 1 IFN), **Validation** **•** Study of the mutant *STAT2* alleles in *STAT2* deficient human cell line, and patient’s immortalized fibroblasts • Patient cells hyper-sensitive to IFN-α → prolonged JAK/STAT signaling/transcriptional activation • Biochemical confirmation that mutant allele is GOF in homozygous, but not heterozygous, combination • Impaired interaction of GOF STAT2 protein with USP18, a negative regulator of type 1 IFN responses	Table 7 Subtable 1	[40, 41]	
***RIPK1*** (12 patients; 5 families, 2 papers)	AD	• Normal T and NK cell numbers • Low/normal CD4^+^ T cells • Normal/hi CD8^+^ T cells • ↑ DN T cells	• Normal B cells	ND	• Autoinflamm disorder: regular/prolonged fevers, lymphadenopathy, spleno/hepatomegaly, ulcers, arthralgia, GI features, • ↑ inflamm markers, ↑ pro-inflamm cytokines/gene signature; • Responsive to Tocilizumab (not IL1/TNF blockade)	Table 7 Subtable 2	[42, 43]	
***NCKAP1L*** (9 patients; 7 families, 3 papers)	AR (LOF)	• Normal T cell numbers • ↑T_CM_, exhausted cells; • Possibly immature NK cells but intact function	• Normal B cells and naïve/memory subsets • ↑ CD21^lo^ cells	• Normal/ ↑ Ig levels • autoAbs	• Recurrent URTI, skin rashes/abscesses, ulcers, • Anti dsDNA Abs, SLE-like, lymphadenopathy, fever, HLH-like • FTT • Immunodeficiency coupled with atopy, lymphoproliferation, hyperinflammation ,and cytokine overproduction (↑ Th1) • ↓ T cell proliferation, cytoskeletal defects;	Table 7 Subtable 3	[44–46]	
***UBA1*** (25 patients)	XL (somatic LOF mutations)	• ↓ Peripheral lymphocyte counts • Loss of immature B cells, nonclassical and intermediate monocyte populations			• Late adulthood onset treatment-refractory inflammatory syndrome • Fevers, cytopenias, dysplastic bone marrow, • Neutrophilic cutaneous and pulmonary inflammation, chondritis and vasculitis • Most patients have an inflammatory syndrome (relapsing polychondritis, Sweet’s syndrome, polyarteritis nodosa, giant-cell arteritis) or a hematologic condition (MDS, multiple myeloma) • Often fatal Dysregulated proinflammatory neutrophil activation, high inflammatory markers in patients’ serum **Validation:** **•** Overexpression of mutant allele favored the production of a catalytically deficient UBA1 • Defective ubiquitylation of patients’ mutant monocytes • CRISPR-Cas9 *Uba1*–deficient zebrafish model recapitulates the phenotype of systemic inflammation	Table 7	[47]	
**Novel phenocopies of inborn errors of immunity**								
**Disease**			**Mechanisms of disease pathogenesis**		**Associated/clinical features**	**Table**	**Refs**	
Severe COVID-19			• High levels of neutralizing autoAbs against type 1 IFNs (primarily IFNα, IFNω)		• Severe, life-threatening infection with SARS-CoV-2	Table 10	[48]	

*Abbreviations*: *AR*, autosomal recessive; *AD*, autosomal dominant; *AID*, activation-induced cytidine deaminase; *CSR*, class switch recombination; *SHM*, somatic hypermutation; *MDS*, myelodysplastic syndrome; *LOE*, loss of expression; *LOF*, loss of function; *GOF*, gain of function; *DN*, dominant negative; *MSMD*, Mendelian susceptibility to mycobacterial disease; *HLH*, hemophagocytic lymphohistiocytosis; *FTT*, failure to thrive; *hPSC*, human pluripotent stem cells; *iPSC*, induced pluripotent stem cells; *CMV*, cytomegalovirus

• Not all Tables in the 2020 classifications [2, 3] are listed in the “Table” column because not all Tables are represented by the new variants detailed above

• Mutations in *PAX1* previously reported to cause OTFCS2 (but CID/SCID not reported) [49]

• Variants in *IL6ST* have been previously listed to cause an IEI due to recessive partial LOF alleles [50]

• Dominant-negative mutations in *IL6ST* all target the intracellular domain of gp130, truncating the STAT3 binding sites as well as recycling motif, leading to sustained expression of a dead receptor (as opposed to recessive alleles, when detected in heterozygous carriers are benign)

• *Fnip1* ko mice also previously reported [12, 13]; humans very similar

• Variants in *CEBPE* have been previously listed to cause an IEI due to recessive LOF alleles [51]

• Mutations in *CDC42* previously identified in individuals with neurodevelopmental delay (Takenouchi-Kosaki syndrome) [52]; in these patients with autoinflammation and *CDC42* mutations no such features were noted, except mild facial dysmorphism in some [33–39]

.• Variants in *STAT2* have been previously listed to cause an IEI due to recessive LOF alleles [53, 54]

• The same amino acid was found to be affected (R148Q/W) for both families affected by *STAT2* GOF [53, 54]

• Variants in *RIPK1* have been previously listed to cause an IEI due to recessive LOF alleles [55, 56]; the heterozygous dominant mutations in *RIPK1* reported here all affect the D324 amino acid residue that is important for cleavage

We also considered (i) the numbers of individuals affected by the novel variants, (ii) sufficient justification for excluding alternative candidate gene variants identified in single cases especially in situations of consanguinity with recessive disease, (iii) the depth of the clinical descriptions of affected individuals, and (iv) the level of immune and mechanistic characterization.

## Novel Causes of Inborn Errors of Immunity

Currently, inborn errors of immunity are listed in 10 tables: Immunodeficiencies affecting cellular and humoral immunity (Table I), Combined immunodeficiencies (CID) with syndromic features (Table II), Predominantly antibody deficiencies (Table III), Diseases of immune dysregulation (Table IV), Congenital defects of phagocytes (Table V), Defects in intrinsic and innate immunity (Table VI), Autoinflammatory diseases (Table VII), Complement deficiencies (Table VIII), Bone Marrow failure (Table IX), and Phenocopies of inborn errors of immunity (Table X). Several of these tables are further partitioned into various subtables (e.g., Table I is split into Subtable 1 [T^−^B^+^ Severe Combined Immune Deficiency (SCID)], Subtable 2 [T^−^B^−^ SCID] and Subtable 3 [CID, generally less profound than SCID]) [2, 3].

Recently-reported gene defects have been found for most categories of inborn errors of immunity, including novel causes of:

- SCID (*PAX1* [5, 6], *SLP76* [7]);
- CID (*MCM10* [8], *IL6ST* [9–11]);
- Predominantly antibody deficiencies (*FNIP1* [14, 15], *PIK3CG* [16, 17], *CTNNBL1* [18], *TNFSF13* [19]);
- Autoinflammatory diseases (*SOCS1* [20–22], *TET2* [23], *CEBPE* [24], *CDC42* [33–39], *LSM11*, *RNU7–1* [32], *STAT2* [40, 41], *RIPK1* [42, 43], *NCKAP1L* [44–46]), *UBA1* (somatic mutations) [47]; and
- Susceptibility to infection with specific pathogens (*MAPK8* [31]; *TBX21* [25], *IFNG* [26], *NOS2* [28], *SNORA31* [29], *ATG4A*, *MAP1LC3B2* [30]) (Table 1).

Notably, several of these genes are already included in previous IUIS updates, namely *IL6ST*, *STAT2*, *CEBPE*, and *RIPK1* [2, 3]. However, they are listed here because the variant identified is pathogenic via a distinct mechanism and/or different mode of inheritance; i.e., autosomal recessive (AR) vs autosomal dominant for *IL6ST* [9] or *RIPK1* [42, 43], partial deficiency vs complete deficiency for *IL6ST* [10, 11], or AR loss of function vs AR gain of function for *CEBPE* [24] or *STAT2* [40, 41]*.* Furthermore, the GOF variants reported for *CEBPE* appear to represent the first described germline neomorphic mutation in inborn errors of immunity where the variant allele has completely novel functions not seen for the wild type gene [24]. Thus, these findings underscore the importance of appropriately interpreting genetic variants identified by next-generation sequencing, not discarding variants of unknown significance simply because they do not match the expected zygosity or clinical phenotype of previously reported studies, and to rigorously validate the impact of novel variants on the function of the encoded protein.

## Joining the Dots with Discoveries of Novel Inborn Errors of Immunity

Many known inborn errors of immunity impact a defined signaling pathway such that mutations in components of these same pathways can represent clinical phenocopies of diseases causes by distinct genetic variants (genetic heterogeneity). In other words, physiological homogeneity can be identified for many genotypes underlying a given phenotype. Classic examples of this are Mendelian susceptibility to mycobacterial disease (MSMD), which results from impaired IFNγ-mediated immunity following exposure to mycobacterial species [58], and herpes simplex virus encephalitis (HSE) resulting from impaired TLR3-mediated anti-HSV1 immunity [59, 60]. Thus, variants in genes affecting the production of IFNγ (e.g., *IL12RB1*, *IL12RB2*, IL23R, TYK2, *IKBKG*, *SPPL2A*, *IRF8*) or cellular responses to IFNγ (e.g., *IFNGR1*, *IFNGR2*, *STAT1*, *JAK1*) result in MSMD in otherwise healthy individuals [58]. Similarly, inactivating mutations in signaling components of the TLR3 signaling pathway (*TLR3*, *UNC93B*, *TRIF*, *TRAF3*, *TBK1*, *IRF3*) underlie HSE due to impaired type 1 IFN-mediated central nervous system (CNS) intrinsic immunity against HSV1 [59, 60].

Recent discoveries have further linked common clinical phenotypes with unique genotypes that converge in a shared pathway. Thus, the non-redundant role of IFNγ-mediated immunity in host defense against mycobacterial infection [58] has been definitively established by the identification of individuals with inactivating bi-allelic mutations in not only *IFNG* itself [26] but also *TBX21* [25], the transcription factor that regulates expression and production of IFNγ.

Interestingly, variants in the small nucleolar RNA *SNORA31* predispose affected individuals to HSE. Mechanistically, patient’s iPSC-derived cortical neurons were found to be highly susceptible to HSV-1 infection in vitro, and this could be restored by exogenous IFNβ [29, 60]. However, responses of these cells to TLR3 and IFNβ, but not HSV1, are intact, revealing that *SNORA31* functions to regulate cell-intrinsic immunity to HSV-1 by a mechanism independent of TLR3 signaling [29, 60]. The discovery of individuals with *SNORA31* variants will facilitate further understanding of CNS-intrinsic host defense.

The discoveries of individuals with complete gp130-deficiency due to null/nonsense bi-allelic mutations of *IL6ST* [11], or pathogenic dominant-negative heterozygous variants of *IL6ST* [9], and a phenotype of eczema, hyper-IgE, and eosinophilia, likely explain these features of autosomal dominant hyper-IgE syndrome due to STAT3 negative dominance [61] and further highlight the role of IL-6 signaling in restraining atopic and allergic responses. Furthermore, the lack of mucocutaneous candidiasis in patients with impaired signaling via receptors for IL-6 (*IL6R*, *IL6ST* mutations [9, 11, 50, 62, 63]; anti-IL-6 autoantibodies [64]), IL-23 (biallelic *IL23R* variants) [65] or IL-21 (biallelic *IL21* or *IL21R* variants) [66] argues that individually these cytokines are not required for the STAT3-mediated generation of human Th17 cells and host defense against fungal infections. Rather, the combinatorial defect of impaired STAT3 signaling downstream of these receptors explains chronic mucocutaneous candidiasis in an individual with dominant-negative *STAT3* mutations. These findings again reveal the capacity for inborn errors of immunity to provide convincing evidence for basic immunological concepts. Indeed, this is further exemplified by the discovery that variants of *ATG4A* or *MAP1LC3B2* cause recurrent HSV2 infection of the CNS, thereby establishing hitherto non-redundant functions of the autophagy pathway in non-hematopoietic cell-mediated intrinsic anti-viral immune responses [30].

## SARS-CoV2 and Inborn Errors of Immunity

The COVID19 pandemic of 2020 has clearly changed the world in many ways. It has also yielded opportunities to understand host requirements for immunity against SARS-CoV2 infection. A recent study of ~ 650 individuals who developed severe COVID-19 found that ~ 3.5% of patients harbored germline loss-of-function variants in genes previously found to be important for host defense against influenza or other viral infections (e.g., bi-allelic loss of function mutations of *IRF7* or *IFNAR1*, heterozygous mutations in *TLR3*, *TICAM1*, *TBK1*, or *IRF3*) [67] due to the key role of these genes in the type 1 IFN signaling pathway [59, 68]. An accompanying study found that, strikingly, ~ 10% of patients with severe COVID-19 have high levels of neutralizing autoantibodies (autoAbs) against type 1 IFNs in their serum [48]. The impact of these autoAbs was evidenced by the inability to detect IFN in serum from these patients, and their capacity to prevent anti-viral immune responses in vitro [48] (Table 1). These studies defined a crucial and non-redundant role for type 1 IFNs in immune control of SARS-CoV2 infection, and thus prevention of severe COVID-19. Furthermore, they also established that autoAbs against type 1 IFN phenocopy an inborn error of immunity, as previously determined for autoAbs against IFNγ and susceptibility to mycobacterial disease, anti-Th17 cytokine (IL-17A, IL-17F, IL-22) autoAbs in individuals with chronic mucocutaneous candidiasis, or pyogenic infections due to anti-IL-6 autoAbs [64, 69].

## Conclusions

Discoveries over the past 12 months in the field of inborn errors of immunity have further identified non-redundant functions of key genes in human immune cell development, host defense, and immune regulation. In some cases, these functions go well beyond what may have been expected or anticipated based on animal models (e.g., *TBX21* [25]). They have also already highlighted the heterogenous phenotypes that can result from variants in the same gene (e.g., *CDC42* [33–39, 52]), indicated that significant diseases can arise from mono-allelic or bi-allelic loss of function (*IL6ST* [9], *RIPK1* [42, 43]) or bi-allelic loss- or gain-of-function (*CEBPE* [24], *STAT2* [40, 41]) variants in the same gene, or from autoAb phenocopies of monogenic lesions (e.g., COVID19 and anti-IFN Abs) [48], and identified novel somatic mutations as pathogenic causes of immune disorders (*UBA1*) [47]. Importantly, they have also provided opportunities for therapeutic interventions, such as JAK inhibitors to treat STAT2 gain of function [40, 41] or SOCS1 deficiency [22], IFNγ to treat mycobacterial disease [25, 26], or early IFN-β or IFN-α2a treatment of SARS-CoV2 infection in COVID-19 patients with autoantibodies against IFN-α or IFN-ω [67] or impaired type 1 IFN responses [70]. This snapshot of genetic discoveries underpinning human immune disorders further highlights the critical contributions of inborn errors of immunity to our broader understanding of basic, translational, and clinical immunology.

## Data Availability

Not applicable.
